# Optical coherence tomography and optical coherence tomography angiography: essential tools for detecting glaucoma and disease progression

**DOI:** 10.3389/fopht.2023.1217125

**Published:** 2023-07-28

**Authors:** Yukihiro Shiga, Takashi Nishida, Jin Wook Jeoung, Adriana Di Polo, Brad Fortune

**Affiliations:** ^1^ Neuroscience Division, Centre de Recherche du Centre Hospitalier de l’Université de Montréal, Montréal, QC, Canada; ^2^ Department of Neuroscience, Université de Montréal, Montréal, QC, Canada; ^3^ Hamilton Glaucoma Center, Shiley Eye Institute, Viterbi Family Department of Ophthalmology, University of California, San Diego, San Diego, CA, United States; ^4^ Department of Ophthalmology, Seoul National University Hospital, Seoul National University College of Medicine, Seoul, Republic of Korea; ^5^ Discoveries in Sight Research Laboratories, Devers Eye Institute and Legacy Research Institute, Legacy Health, Portland, OR, United States

**Keywords:** glaucoma, optical coherence tomography, optical coherence tomography angiography, detection, progression

## Abstract

Early diagnosis and detection of disease progression are critical to successful therapeutic intervention in glaucoma, the leading cause of irreversible blindness worldwide. Optical coherence tomography (OCT) is a non-invasive imaging technique that allows objective quantification *in vivo* of key glaucomatous structural changes in the retina and the optic nerve head (ONH). Advances in OCT technology have increased the scan speed and enhanced image quality, contributing to early glaucoma diagnosis and monitoring, as well as the visualization of critically important structures deep within the ONH, such as the lamina cribrosa. OCT angiography (OCTA) is a dye-free technique for noninvasively assessing ocular microvasculature, including capillaries within each plexus serving the macula, peripapillary retina and ONH regions, as well as the deeper vessels of the choroid. This layer-specific assessment of the microvasculature has provided evidence that retinal and choroidal vascular impairments can occur during early stages of glaucoma, suggesting that OCTA-derived measurements could be used as biomarkers for enhancing detection of glaucoma and its progression, as well as to reveal novel insights about pathophysiology. Moreover, these innovations have demonstrated that damage to the macula, a critical region for the vision-related quality of life, can be observed in the early stages of glaucomatous eyes, leading to a paradigm shift in glaucoma monitoring. Other advances in software and hardware, such as artificial intelligence-based algorithms, adaptive optics, and visible-light OCT, may further benefit clinical management of glaucoma in the future. This article reviews the utility of OCT and OCTA for glaucoma diagnosis and disease progression detection, emphasizes the importance of detecting macula damage in glaucoma, and highlights the future perspective of OCT and OCTA. We conclude that the OCT and OCTA are essential glaucoma detection and monitoring tools, leading to clinical and economic benefits for patients and society.

## 1 Introduction

Glaucoma, the leading cause of irreversible blindness worldwide, is characterized by the progressive death of retinal ganglion cells (RGCs), resulting in spatially distinct patterns of visual field (VF) loss that correspond to bundle patterns of RGCs axons (1). Glaucoma is a multifactorial disease, and elevated intraocular pressure (IOP) is a major modifiable risk factor for disease development and progression (2, 3). In addition to other risk factors such as older age and myopia (4, 5), vascular dysfunction has been reported to play a crucial role in the disease process (6, 7), as supported by recent genome-wide association study (8). The number of people with glaucoma worldwide was estimated at 80 million in 2020, and to surpass 100 million in 2040 with an aging global population (9). Glaucoma and other vision-threatened diseases affect the increased risk of fractures, depression, and impairments in daily living, resulting in decreased quality of life (QOL) in patients, as well as contributing to the global economic burden (10, 11). Thus, early detection of glaucoma and disease progression is a key strategy to provide timely therapeutic intervention to slow disease progression, leading to clinical and economic benefits.

Optical coherence tomography (OCT) imaging currently plays a central role in diagnosing and managing glaucoma (12–14). OCT allows non-invasive, objective quantification of changes in structures critical to the diagnosis and pathology of glaucoma, from the anterior segment of the eye, such as the iridocorneal-angle, to the posterior segment, including the retinal nerve fiber layer (RNFL), macula, and optic nerve head (ONH). Advances in OCT technology have led to dramatic increases in the scan speed, reduced acquisition time, enhanced image quality, improved segmentation accuracy and diagnostic algorithms, all of which contribute to more accurate and reproducible measurements for early diagnosis and refined monitoring of glaucoma. Moreover, the technological innovation of OCT has enabled much greater visualization of deeper structures within the ONH thought to be critically important to glaucoma pathophysiology, such as the lamina cribrosa (LC); although, quantitative morphometrics of the LC (such as its depth, shape or microarchitecture, pore size, beam diameter, etc.) have not yet achieved widespread clinical application for glaucoma diagnosis and monitoring (15).

More recently, OCT angiography (OCTA) has emerged as a technique for noninvasive assessment of the microvasculature within the superficial, intermediate and deep capillary plexuses of the posterior pole and macula, as well as the radial peripapillary capillary plexus and ONH microvasculature (16–18). Existing commercial instrumentation for OCTA provides quantitative indices of patent capillary beds within each plexus, such as vascular density. Evidence from studies using OCTA has accumulated to demonstrate that abnormalities of retinal and choroidal vascular systems occur in glaucomatous eyes, perhaps even during early stages of disease development.

In this review, we initially describe the normal anatomy of the retina and ONH in order to provide a common framework for understanding glaucomatous changes revealed by OCT and OCTA. Then, we evaluate the utility of OCT and OCTA for glaucoma diagnosis and for detection of its progression. Next, we emphasize the importance of detecting glaucomatous damage to macula, including the papillomacular bundle of axons, which subserves central vision that is so important to a wide variety of daily living tasks and QOL. Finally, we provide updated findings of LC imaging and highlight the future directions of OCT and OCTA. Our review supports the clear conclusion that OCT and OCTA are vital tools for early glaucoma diagnosis and detecting its progression.

## 2 Anatomy of the retina and the ONH relevant to OCT/OCTA detection of glaucoma

### 2.1 OCT visualizes detailed ocular anatomy

**Figure 1:**
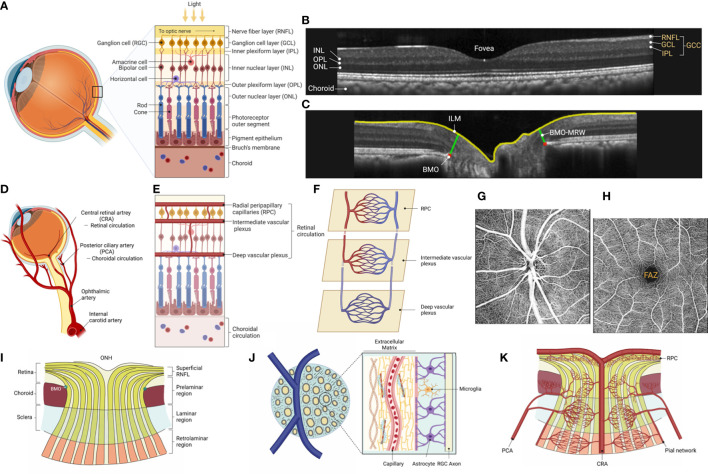
The anatomy of the retina and the ONH. **(A)** External light stimuli are first transmitted to photoreceptor cells and then to the RGCs via bipolar cells, which are interneurons, and the neurons transmit visual information to the brain. The signal transduction from photoreceptor cells to the RGCs is modulated by horizontal and amacrine cells, inhibitory interneurons, at each stage of the retinal neural circuit, subdividing the visual information transmitted to the brain. **(B)** SD-OCT visualizes the three-dimensional anatomy of the retina with nearly cellular scale resolution. OCT cross-sectional images known as B-scans depict two dimensions typically revealing three layers comprised of neuronal cell bodies (outer nuclear layer [ONL], inner nuclear layer [INL], and ganglion cell layer [GCL]), two synaptic layers (outer plexiform layer [OPL] and inner plexiform layer [IPL]), and the axon layer of RGCs (nerve fiber layer [RNFL]). Notably, the IPL, which contains the dendrites of the RGC, the GCL, which consists of the cell bodies of the RGC, and the RNFL, the axon of the RGC, together form the combined retinal layer known as ganglion cell complex (GCC), which is preferentially affected in glaucoma with sparing of deeper retinal layers generally. **(C)** ONH radial scans by SD-OCT. The RGCs project their axons from BMO to the brain. Recently, BMO-MRW (Bruch’s membrane opening minimum rim width), defined as the distance from the anatomical feature defining the outer edge, BMO, to the closest point along the inner limiting membrane (ILM), has been demonstrated to be a reproducible measure of the ONH neuro-retinal rim thickness useful for early glaucoma detection. **(D)** The mammalian retina has two vasculature systems, the choroidal and retinal vasculature, which supply oxygen and nutrients from the ophthalmic artery, a branch of the internal carotid artery. The choroidal circulation delivers oxygen to the outer retina (consumed by photoreceptors and retinal pigmented epithelium, RPE), while the retinal vasculature nourishes the remaining inner retina, including the INL, IPL, GCL, and RNFL. **(E)** The central retinal artery branches into the superior and inferior arcades temporally around the macula, and to the superior and inferior nasal retina, into finer arterioles culminating in a three-layered capillary network of superficial, intermediate and deep vascular plexuses, with an additional plexus known as the radial peripapillary capillary (RPC) plexus serving the thickest parts of the RNFL near the ONH. **(F)** The RPC plexus is the most anterior layer of capillaries lying within the RNFL where it is thickest, which along with the major vessels and the capillaries of the superficial vascular plexus of the GCL form the superficial vascular complex. The intermediate and deep vascular plexuses, respectively, run along the anterior and posterior aspects of the INL; the three capillary networks are interconnected and anastomosing (19). **(G, H)** OCTA provides detail of the microvasculature of each vascular network. Example of en face axial OCTA scans of parapapillary and macular regions in an adult rhesus monkey. The superficial vascular plexus, including RPC plexus, is shown in both images. Note that in the macula, which is highly specialized for central vision, a foveal avascular zone (FAZ) of 450 to 600 mm in diameter is formed, bordered by capillaries running in the inner retinal layers. **(I)** The ONH is a dynamic structure with approximately 1.2 million RGC axons converging and exiting the eye through the inner (BMO) and outer (scleral area) portions of the neural canal. Histologically, the ONH is divided into three layers: superficial RNFL followed by prelaminar region, laminar region, and retrolaminar region. In the superficial RNFL, unmyelinated axons originating from the RGC radiate toward the ONH, enter the ONH and bend and angle to form the optic nerve. In the prelaminar region, RGC axon fibers are bundled in glial columns composed of astrocytes. In the laminar region, the bundled axons pass through a three-dimensional network of collagenous beams called lamina cribrosa. Optic nerve fibers become myelinated by oligodendrocytes in the retrolaminar region. **(J)** Lamina cribrosa, a pivotal site of axonal damage in glaucoma, contains capillary-embedded connective tissue (beams) and glial cells such as astrocytes. Importantly, there is little direct blood supply to the axons within the lamina region. Therefore, the axons within the lamina cribrosa require diffusion of nutrients from the laminar capillaries across to the laminar beams and glial cells to adjacent axons. **(K)** RGC axonal transport is energy-dependent and requires a supply of nutrients to meet its energy demands. Blood flow within the ONH is well-autoregulated, and the vascular supply to the ONH is from CRA and CAs. The superficial RNFL receives its blood supply mainly from RPC derived from the CRA. In contrast, the prelaminar, laminar and retrolaminar layers are supplied primarily by CAs, where the branches of CAs penetrate to those regions through the scleral flange. The capillaries ramified from central retinal and CAs form a dense network in the ONH without clear separation between the four layers. The retrolaminar region is mainly supplied by the CRA and the pial network derived from the CAs. **(A, D, E, F, I, J, K)** generated with BioRender (https://biorender.com/).

Light entering the eye is detected by photoreceptors in the deepest layer of the neural retina, which convert this energy into an electro-chemical signal. The signal is then transmitted across the first synapse to bipolar cells, with lateral inhibitory interactions contributed by horizontal cells in the outer plexiform layer (**Figure 1A**). The bipolar cells convey these signals along to the RGCs via synapses in the inner plexiform layer (IPL), where lateral inhibitory interactions are provided by different classes of amacrine cells. Each RGC extends an unmyelinated axon along the inner most layer of the retina, the RNFL, which then converge to exit the eye through the scleral canal to become the optic nerve. The scleral canal is bridged by a structure known as the LC, comprised of interconnected collagenous beams primarily oriented in a lateral (transverse) plane, forming pores through which the bundles of axons pass. The ONH refers to the intraocular portion of the optic nerve, comprised of the RGC axons, glia (astrocytes and microglia), vasculature, and extracellular matrix (most prominently the LC), as well as the immediately adjacent peripapillary tissues (see Section 6 for more details). At the posterior aspect of the LC, axons become myelinated resulting in a dramatic expansion of the diameter of the optic nerve as it exits the eye and traverses the bony orbit destined for the optic chiasm, where approximately 60% of RGC axons from the nasal retina of each eye cross to the contralateral hemisphere of the brain to join uncrossed fibers from the temporal retina of the contralateral eye and become the optic tract. Each RGC axon will form synapses within specific midbrain targets, primarily the lateral geniculate nucleus of the thalamus, but also the superior colliculus, pretectal nucleus, suprachiasmatic nucleus and other midbrain centers. Most RGCs convey information necessary for image formation, while others are involved in circuits primarily influencing pupillary light reflexes, circadian rhythms and other fundamental processes of the visual system. Thus, death of RGCs and degeneration of the optic nerves result not only in loss of vision, but also disruption of other basic physiological functions such as diurnal rhythms (20).

Continued advances in OCT technology allow detailed visualization of ocular anatomy, including high-resolution cross-sectional views of retinal layers and structural relationships within the ONH. With higher OCT scan speeds, denser scan patterns also provide high resolution *en face* views of any desired layer/structure. For example, the IPL, where dendrites of the RGCs – consisting of at least 17 different subclasses in the primate retina (21) – receive excitatory and inhibitory synaptic inputs from bipolar and amacrine cells. The RGCs project their axons along arcuate paths within the RNFL to the ONH, pass through the inner Bruch’s membrane opening (BMO) en route to the optic chiasm and brain centers where their synaptic contacts are made. The BMO is useful as a landmark to indicate the transition from retina to ONH and to help parameterize OCT for quantitative assessment of ONH structure and glaucoma diagnostics (22) (**Figures 1B, C**).

The central retina, including the highly specialized fovea of the human (and other primate) eye, is dominated by cone photoreceptors, while the more peripheral retina beyond 20 degrees is rod-dominant. The central retina has a considerably thicker IPL and ganglion cell layer (GCL) than the peripheral retina. This is due to the increased density of RGCs in the cone-dominant central retina than in the rod-dominant peripheral retina. Notably, the thickness measurements often used by OCT combining the GCL with IPL, or of the three innermost retinal layers (NFL + GCL + IPL) are referred to as GCIPL and ganglion cell complex (GCC), respectively, which are preferentially affected in glaucoma (22–24) (**Figure 1B**).

### 2.2 OCTA visualizes different layers of the retinal capillary network

Two separate vascular systems supply oxygen and nutrients to the human retina: the choroidal and the retinal vasculatures, which are all derived from the ophthalmic artery, a branch of the internal carotid artery (**Figure 1D**). The choroidal circulation supplies primarily the metabolic needs of the outer retina, particularly the photoreceptors whose mitochondrial-rich inner segments consume virtually all oxygen diffusing from the choroid beyond the retinal pigmented epithelium (RPE), while the retinal vasculature nourishes the remaining inner retina including IPL, GCL, and RNFL (25).

The central retinal artery (CRA) primarily supplies the retinal vasculature, which emerges at the ONH, while in some individuals there may also be cilioretinal arteries arising from the choroid to nourish the central retina (26). The CRA has four main branches extending outward from the ONH each of which supplies one quadrant of the retina, respectively (27, 28). The retinal arterial branches then serve three layers of main capillary networks throughout most of the retina; the superficial vascular plexus, the intermediate capillary plexus, and the deep capillary plexus (19, 29, 30) (**Figures 1E, F**). In addition, the radial peripapillary capillaries (RPCs) comprise a fourth plexus around the ONH, which extends along the arcades and is thought to nourish the additional metabolic needs of the thick RNFL in these arcuate regions (31). Similarly, at the rim of the fovea, where the macula is thickest, a fourth, superficial layer of capillaries can be appreciated using OCTA (30). OCTA non-invasively provides detailed information on the microvascular structures in each vascular network by detecting the variance of reflectivity caused by motion of blood cells within vessels (**Figures 1G, H**). The RPCs are the most superficial layer of capillaries lying within the NFL where it is thickest: at the poles of the ONH and extending along the arcuate bundles toward the macula. The superficial vascular plexus lies deeper (GCL through anterior IPL) and is more widely distributed across the retina than the RPC plexus. Deeper still are the intermediate and deep vascular plexuses, respectively, straddling the anterior and posterior aspects of the inner nuclear layer (INL). Confocal microscopy studies in ex vivo specimens have shown that the intermediate vascular plexus is located in the region close to the bipolar cells in the inner INL. In contrast, the deep vascular network localizes to the outer INL (32). The three capillary networks are interconnected and anastomose with each other, while the deep vascular plexus is “venous side only”, i.e., it does not receive blood flow directly from retinal arterioles, rather it receives flow via anastomoses within the intermediate plexus, then flow from there is one way out only, directly to venules (33, 34) (**Figure 1F**). In contrast, the fovea in the macula, a highly specialized region for central vision, exhibits a capillary-free zone 450-600 mm in diameter, known as the foveal avascular zone (FAZ) within the ring of peri-foveal capillaries running in the inner retinal layers (**Figure 1H**). The blood leaving the capillary beds joins venules, which flow into the major venous branches and leave the eye as the central retinal vein through the middle of the LC, within a common sheath of the CRA.

The choroid is one of the most highly vascularized structures in the body of mammals, accounting for about 85% of the total blood flow serving the retina, in large part to meet the high metabolic demands of the outer retina including photoreceptors and RPE (35) (**Figures 1D, E**). The ciliary arteries (CAs) supply the choroidal vasculature (36) (**Figures 1D, E**). These arteries branch into large-sized blood vessels in the outer choroid, known as Haller’s layer, then into medium-sized blood vessels in Sattler’s layer, which in turn feed the choriocapillaris, the highly anastomosed and fenestrated network of lobule-shaped capillary loops adjacent to Bruch’s membrane. The medial and lateral paraoptic short posterior CAs converge toward the ONH to form an oval-shaped anastomotic circle, the circle of Zinn-Haller, considered the main arterial supply to the LC (37). The ring of Zinn-Haller is incomplete in 23% of cases and complete in 33% of individuals but with some areas of narrowing (38). Such anatomic variations may make this part of the ONH circulation particularly vulnerable to ischemia. The venous drainage of the choroid is through three to eight (typically four) vortex veins that drain into the superior and inferior ophthalmic veins.

The choroidal and retinal circulations differ not only morphologically but also physiologically due to differences in their autonomic innervations and capacity for autoregulation (39). The choroidal vessels are innervated and regulated by both sympathetic and parasympathetic nerves and are less well autoregulated, while the retinal vasculature lacks autonomic innervation; instead, it is tightly controlled by autoregulatory mechanisms, involving astrocytes, pericytes, vascular smooth muscle cells and endothelial cells.

### 2.3 Axons, connective tissue, and glial framework in the ONH

The ONH has a complex architecture to accommodate dynamic processes including fluctuations of blood flow and biomechanical loads, while maintaining stability for the approximately 1.2 million RGC axons that converge there to exit the eye through the inner (BMO) and outer (scleral) portions of the neural canal (40) (**Figure 1I**). Axons of RGCs transport molecules, vesicles, and organelles in both directions to maintain neuronal function. Histologically, the ONH is divided into three layers: the superficial RNFL followed by the prelaminar region, the lamina region, and the retrolaminar region.

In the RNFL, the unmyelinated axons originating from RGCs run radially to converge at the ONH, bending posteriorly there to leave the eye through the scleral canal to become the optic nerve. The RNFL contains the RGC axons supported by both astrocytes and Müller glia, which separate groups of axons into bundles and whose processes penetrate the bundles to wind between individual axons. Optic nerve fibers are supported by astrocytes, which surround the nerve fiber bundles within the ONH, and extend processes into the center of bundles. In the prelaminar region, optic nerve fibers are bundled by glial columns composed of astrocytes (41). In the scleral portion, the bundled axons pass through a three-dimensional network of connective tissue beams known as LC. Most laminar LC beams contain a capillary at their core and are lined by astrocytes (**Figure 1J**). The ONH is constantly subjected to IOP, intracranial pressure, and optic nerve traction during eye movement, and the LC is thought to be the pivotal site for such mechanical stress-induced axonal and connective tissue damage in glaucoma (42–44). The retrolaminar region begins at the posterior aspect of the LC, where optic nerve fibers become myelinated by oligodendrocytes and the nerve is surrounded by the dural sheath. The connective tissue of the LC is contiguous with the septae within both retrolaminar and prelaminar regions, comprised of glia and extracellular matrix, as well as with the vascular adventitia. The lateral insertions of the LC collagen fibrils are primarily into the sclera, but can also insert into the pia and are thought to remodel as a result of glaucoma (45, 46). Continuous advances in OCT, including swept-source OCT (SS-OCT) approaches, enable greater visualization and quantitative evaluation of the structural changes of LC during glaucomatous damage, such as LC depth and changes in the shape and size of the lamina pores.

### 2.4 Vascular supply to the ONH

RGC axonal transport is energy-dependent and requires a supply of nutrients to meet its energy demands (47). Although the detailed mechanisms by which nutrients are delivered to axons within the ONH remain incompletely understood, efficient vascular perfusion is considered a critical requirement for active axonal transport within the ONH. Blood flow within the ONH is well-autoregulated, and the vascular supply to the ONH is from CRA anteriorly and CAs posteriorly, with these capillary beds being highly anastomotic (48) (**Figure 1K**).

As mentioned above, the superficial RNFL and prelaminar layer receive its blood supply mainly from RPCs derived from the CRA. In contrast, laminar and retrolaminar layers are supplied primarily by CAs, where the branches of CAs penetrate to those regions through the scleral flange. The capillaries ramified from CRA and CAs form a dense network in the ONH without clear separation between the four layers (48). Importantly, there is little direct blood supply to the axons within the lamina region (47) (**Figure 1J**). Thus, axonal nutrition within the lamina primarily relies on the diffusion of nutrients from the laminar capillaries, across the lamina beam and the astrocytes, to the adjacent axons (49). The retrolaminar region is mainly supplied by the CRA and the pial network derived from the CAs. Venous drainage of the ONH occurs primarily through the central retinal vein.

## 3 OCT in glaucoma

### 3.1 Generations of OCT

OCT is a noninvasive *in vivo* imaging technique that uses interferometry of low-coherence light to capture depth-resolved reflectance of retinal structures (50). Currently, at least the third generation of OCT is commercially available. In general, scan speed and image quality have improved with each generation, as has the signal-to-noise ratio (SNR) and resolution with increasing depth into the tissue sample.

Time-domain OCT (TD-OCT) was the first generation of OCT, in which the axial depth of near-infrared light backscattered from the retina is determined by comparison to the round-trip optical path length of a reference beam that is mechanically modulated by physical displacement of a mirror. Thus, the reflectance at each axial (depth) position must be determined in sequence by movement of the reference mirror in order to reconstruct the longitudinal reflectivity profile (known as an “A-scan” or “A line”) at each lateral pixel position. The need for axial scanning in the depth direction, limited by the maximum oscillation speed of the reference mirror, meant that, in practice, the maximum scanning speed of commercially available TD-OCT was 400 A-scans/second (51). This A-line acquisition rate with a grid scan pattern of 200 x 200 A-lines would require nearly two minutes to complete. This relatively slow rate is also more likely to produce artifacts related to eye movements and blinks occurring during the scan.

SD-OCT is the second generation of OCT typically using a near-infrared source with a center wavelength of approximately 850 nm and is a type of Fourier domain OCT (FD-OCT) (52, 53). SD-OCT, unlike TD-OCT, uses Fourier analysis of the spectrally-resolved interference pattern or spectral interferogram created by the interaction of light backscattered from the retina with light reflected from the reference mirror to simultaneously reconstruct the entire reflectivity profile in depth (i.e., the full A-line) without the need for mechanical displacement of the reference mirror and with greater SNR. This simultaneous evaluation of all axial depth samples comprising each A-scan with the SNR gain significantly increases the scanning speed to 100,000 or more A-scan/s for current commercial devices. In addition to reducing acquisition times, SD-OCT has dramatically improved SNR and image quality. However, the signal roll-off inherent to SD-OCT, i.e., reduction of signal strength with increasing depth, is an important limitation of this approach (54).

SS-OCT, another type of FD-OCT, enables high-speed scanning of 200,000 or more A-scan/s on current commercial devices (55). SS-OCT uses a narrowband laser that sweeps a wide frequency bandwidth at high speed. Temporally encoded spectral interferograms are captured by a high-speed photodetector, eliminating the need for a spectrometer. With a central wavelength of approximately 1050 nm, SS-OCT enables high-speed scanning and imaging of deeper ocular structures, such as LC, owing to a negligible signal roll-off and less scattering compared to near infrared sources. This is offset slightly by the lower resolution corresponding to the longer wavelength.

### 3.2 Glaucoma diagnosis using OCT

SD-OCT is a powerful tool for diagnosing glaucoma, dramatically increasing over previous technologies our ability to quantitatively measure parameters of structural integrity, including the circumpapillary RNFL (cpRNFL) thickness, macular inner retinal thickness, ONH neuroretinal rim thickness and even aspects of deeper ONH structures such as LC depth and curvature. Although SD-OCT-derived measurements generally show excellent diagnostic accuracy, detection of glaucomatous damage in the earliest stages, and in eyes with concurrent high myopia, pose continued challenges.

**Figure 2:**
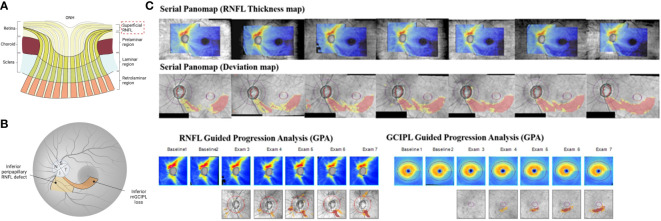
Utility of OCT scans for glaucoma diagnosis and disease progression detection. **(A)** The hallmark of glaucoma is characterized by the progressive thinning superficial RNFL. **(B)** In the peripapillary RNFL scan, average RNFL thickness and inferior-sector RNFL thickness, the susceptible site against glaucomatous damage, are widely used as reliable diagnostic and progression detective OCT parameters. In the macula scan, GCIPL, in particular, is effective in distinguishing glaucomatous eyes from healthy eyes and in detecting disease progression across a wide range of glaucoma stages, including advanced stages of glaucoma due to less floor effect. The abnormal macular RGC thickness is often detectable from earlier stages of the glaucomatous eye compared to RNFL measurements, while the progression of peripapillary RNFL thinning sometimes precede macular GCIPL thinning. Thus, measurements of peripapillary RNFL and GCIPL are complementary and integrating the two parameters can provide additional information on early detection of disease deterioration. **(C)** Serial combined wide-field OCT maps (Panomaps) and Guided Progression Analysis (GPA) results for retinal nerve fiber layer (RNFL) and ganglion cell-inner plexiform layer (GCIPL) of an early glaucomatous eye. Structural progression on the serial Panomaps (RNFL thickness map, deviation map set) and in both RNFL and GCIPL GPAs were clearly detected. (JAMA Ophthalmology 2018:136:1121-1127. Originally published by and used with permission from American Medical Association.) **(A, B)** generated with BioRender (https://biorender.com/).

Several studies investigating the performance of OCT in glaucoma have shown promising results. In the case of cpRNFL thickness measured by SD-OCT, parameters of global average cpRNFL thickness and inferior-sector average cpRNFL thickness, which is the most susceptible site to glaucomatous damage, are widely used because they are most accurate for diagnostic purposes (sensitivity: 60-98% and specificity: 80-95%, respectively) (56–60) (**Figures 2A, B**). Recent studies have also shown that the mean cpRNFL thickness parameter can detect glaucomatous damage even well before VF defects appear. For example, a recent study demonstrated that the percentage of eyes with abnormal average cpRNFL thickness four years before VF defect detection was as high as 35%, and 19% of eyes showed abnormalities eight years prior (61), suggesting the usefulness of cpRNFL measurements for early glaucoma detection. In patients with preperimetric glaucoma, the sensitivity of the OCT parameters ranged from 21.0% to 87.1%, with the criterion of abnormal at the 5% level; the probability of the measured cpRNFL thickness being within the normal range for the age-matched population (62). It should be noted, however, that the cpRNFL thickness is susceptible to individual ONH structural variabilities, such as tilted disc and peripapillary atrophy that are frequently seen in highly myopic eyes.

In contrast, macular parameters are superior in yielding more consistent images with less structural variation between individuals, at least when there is no concurrent macular/retinal disease (63). Segmentation of the macular inner retinal layers allows for layer-specific quantification of macular RGC damage, such as macular RNFL, GCIPL, and GCC including macular RNFL, GCL and IPL. Among macular parameters, GCIPL and GCC, in particular, are effective in distinguishing glaucomatous eyes from healthy eyes across a wide range of glaucoma stages, including preperimetric glaucoma (23, 64, 65).

Myopic eyes have various optic disc shapes such as disc tilt, peripapillary atrophy, and posterior staphyloma. These structural variations make the diagnosis of glaucoma challenging in myopic eyes. Macular structure is known to be less affected by the degree of myopia and myopia-related optic disc change than cpRNFL thickness. Therefore, macular parameters are effective in detecting glaucoma in highly myopic eyes. Notably, a previous study has shown that the inferotemporal GCIPL thickness is the best parameter for glaucoma detection in myopic eyes (66) (**Figure 2B**). Furthermore, the asymmetry of structural damage in early glaucoma is noteworthy: automated detection of hemifield differences across the horizontal raphe on the GCIPL thickness map can identify early structural damage with high reproducibility (67). This analysis is particularly effective in cases of high myopia with tessellated fundus, where RNFL defects cannot be clearly observed. Additionally, each commercial OCT software has their own respective normal eye databases; however, using deviation maps in highly myopic or highly hyperopic eyes can pose challenges. The application of software with a database of high myopia (RS-3000 SD-OCT; Nidek, Gamagori, Aichi, Japan) has been reported to enhance the diagnostic specificity of glaucoma (68).

Due to faster scanning speeds, recent SS-OCT devices enable covering a larger area in a single scan compared to performing separate scans of the optic disc and macular areas. Wide-field scans can comprehensively capture structural changes from the peripapillary ONH to macular regions in a single volume, thus allowing excellent visualization of the distal temporal margin of RNFL defects and for objective evaluation of early glaucomatous changes (69). Recent studies showed that the wide-field RNFL thickness map using SS-OCT performed well in distinguishing eyes with preperimetric and early glaucoma from healthy eyes (70, 71). The wide-field RNFL map of SS-OCT can be a useful tool for early detection of glaucoma.

Several ONH parameters can be obtained from OCT 3D volume scan data, including rim thickness or width, rim area and cup-to-disc ratio. Recently, Bruch’s membrane opening minimum rim width (BMO-MRW), defined as the minimum distance from the anatomical outer edge, BMO, to the inner limiting membrane, has been proposed as a sensitive, reproducible measurement of the ONH for early glaucoma detection (72). It has also been shown that 3D neuroretinal rim thickness, defined as the distance between the BMO and the vitreoretinal interface, helps reduce false positives in glaucoma diagnosis and improves the accuracy of glaucoma detection in myopic eyes (73).

Given that structural damage in glaucoma may only be revealed by a single diagnostic parameter, integrating and interpreting multiple pieces of information obtained from cpRNFL, macular, and ONH scans may improve the sensitivity of glaucoma detection (**Figure 2B**) (74, 75).

### 3.3 Detection and monitoring glaucoma progression using OCT

The establishment of a highly sensitive method for assessing glaucoma progression is crucial for appropriate therapeutic intervention to delay irreversible RGC death and preserve vision. Numerous studies have confirmed the reliability of OCT in detecting glaucoma progression; however, a multitude of factors can affect its predictive accuracy, such as the variability of disease progression, glaucoma severity, and age-related changes.

Glaucoma progression algorithms using SD-OCT perform event-based or trend-based analysis. The event-based analysis (e.g., Guided Progression Analysis for both macula and ONH [GPA] in Cirrus OCT [Carl Zeiss Meditec Inc., Dublin, CA]) considers progression when follow-up measurements exceed a predetermined threshold identifying significant change relative to the baseline. On the other hand, the trend analysis detects progression by evaluating the linear rate of change of parameters measured over time. Each approach has its limitations; the event-based analysis may misread outliers as “progression,” while trend-based analysis requires multiple observations to be reliable. Both are subject to confounding effects of natural age-related change, which must be considered and distinguished from significant glaucoma progression (76). Medeiros et al. reported that a Bayesian modeling approach based on functional and structural data improves sensitivity and specificity in detecting glaucoma deterioration compared to standard linear regression modeling approaches (77). Thus, there is room for improvement in optimizing mathematical models in progression detection. Measurements of cpRNFL and macular GCIPL have been widely used in the algorithms for glaucoma progression detection with excellent long-term reproducibility (78).

A longitudinal SD-OCT study of glaucomatous and healthy eyes followed for three years found that glaucoma progressors determined by fundus photographs had a significantly higher rate of cpRNFL loss than non-progressors (79). Although mean cpRNFL thickness is the primary parameter for detecting structural progression in glaucoma, RNFL thickness in the inferior quadrant or inferior temporal sector is generally the best predictor of progression (80, 81). However, a limitation of cpRNFL evaluation for tracking progression is that it is less sensitive than VF testing in advanced cases due to a ‘floor effect’ that occurs when the remaining tissue thickness reaches the lower limit of measurable change (82–84). Two longitudinal studies that followed advanced glaucomatous eyes for an average of 2.2 to 5 years reported no significant differences in the rate of changes in mean cpRNFL thickness between VF progressors and non-progressors (85, 86). Therefore, it may not be possible to detect widespread cpRNFL thinning in the eyes with advanced glaucoma (although focal thinning is often still appreciable in sectors with some preserved RNFL tissue). In addition, a prospective study evaluating age-related cpRNFL thinning showed that cpRNFL thickness decreases with age by as much as -0.52 μm/year in healthy eyes (87), suggesting that age-related structural loss should be considered when using cpRNFL thickness to determine glaucoma progression.

In contrast, evaluation of GCIPL may be more sensitive in detecting progression regardless of severity because the floor effect is less likely to occur (88). The GCIPL thinning rate on OCT was significantly faster for patients with glaucoma progression than for those without progression, suggesting that trend-based analysis of GCIPL thickness on OCT may be useful for assessing glaucoma progression objectively and quantitatively (89). A longitudinal study of eyes with advanced glaucoma determined by visual field testing showed that during a mean follow-up period of 2.2 years, the decrease in macular GCIPL thickness was significantly greater in the progressive group than the non-progressive group but not in cpRNFL thickness (86). These results indicate that macular thickness evaluation is more applicable for detecting disease progression in advanced glaucoma. Furthermore, a study investigating the temporal-spatial relationship between inferior macular GCIPL defects and corresponding peripapillary RNFL defects on the OCT deviation map revealed that in early glaucomatous eyes, the detected GCIPL changes were often prior to the RNFL change (90). These findings suggest that the GCIPL deviation map is sensitive and can detect macular abnormalities from early on. Conversely, the progression of cpRNFL thinning may precede macular GCIPL thinning (91), indicating that measurements of cpRNFL and GCIPL are complementary and integrating the two parameters can provide additional information on early detection of disease deterioration (**Figure 2C**). Indeed, for the slow time course of progression in most patients, and the inter-visit duration typically followed in clinical glaucoma management, loss of RGC soma and axons, and thus of macular inner retinal and cpRNFL tissue should correspond, with discrepancies being due largely to limitations of detection by clinical instrumentation and standard diagnostic protocols.

Due to this complementary aspect of the macular and peripapillary regions, wide-field SS-OCT imaging has the potential to facilitate structural progression detection by providing comprehensive and extensive measurements of both structures. In a study of early stages of glaucomatous eyes (mean VF mean deviation, -1.9 dB), serial use of Cirrus high-definition OCT wide-field integrated ONH-RNFL/macula-GCIPL maps showed well-discriminating structural progression within a minimum 3-year follow-up period (92) (**Figure 2C**). Moreover, Hood et al. have recently developed a one-page report that provides a topographic overview of the ONH-RNFL/macula-GCIPL probability maps acquired by wide-field SS-OCT scanning with VF points (69, 93). It is expected that the actual static automated perimetry 24-2 and 10-2 VF information of the patient will be superimposed into this report to visualize the structure-function relationship in glaucoma and become a valuable tool for predicting disease progression. Further longitudinal studies with longer follow-up periods are needed to fully understand the clinical utility of wide-field SS-OCT for detecting glaucoma progression. In addition to the ONH-RNFL/macula-GCIPL thickness, only in cases with focal RNFL defects, a region-of-interest approach that measures the enlargement of RNFL defect width effectively detects progression, as the corresponding ONH region is most likely to progress (94).

Beta-zone peripapillary chorioretinal atrophy (beta-zone PPA) is an area of the visible sclera adjacent to the clinically visible disc margin. It corresponds to a region without RPE and is frequently seen in myopic eyes. Recent imaging studies have demonstrated that the ONH OCT B-scan can identify two new subsets of beta-zone PPA (also called beta-zone PPA, an area with Bruch’s membrane, and gamma-zone PPA, a site free of Bruch’s membrane) (95). In two observational studies of POAG eyes followed for more than 2 years, POAG eyes with beta-zone PPA showed faster structural and functional loss progression than those with no PPA or gamma-zone PPA (96). Thus, the new classification of beta-zone PPA by OCT findings may provide new insights into predicting glaucoma progression.

## 4 OCTA in glaucoma

### 4.1 OCTA

OCTA is a non-invasive, dye-free technique for three-dimensional imaging of the retinal and choroidal microvasculature *in vivo* (97). OCTA is based on detecting variability of local reflectance signals, caused primarily by the motion of red blood cells through the blood vessels within the tissue sample (97). Either by detecting decorrelation over time or from different parts of the reflected source spectrum, OCTA scans enable visualization and some degree of quantification of microvascular perfusion; however, it should be noted that each commercially available OCT device uses its unique algorithm for detecting, representing and analyzing OCTA signals and microvascular perfusion (98).

Current OCTA is capable of scanning the optic disc and macula area, and in the case of wide-field scan types, the entire posterior pole in a single scan. OCTA scans of the optic disc region are commonly performed with a volumetric (grid pattern) scans centered around the optic disc. In contrast, the macular OCTA scan is conducted with volumetric scans centered on the fovea. The segmentation of retinal layers using the images obtained from the optic disc and the macular scans, followed by the extraction of vascular images at the targeted retinal layer, such as en face projections of a selected slab with determined depth and thickness, allows the observation of the retinal capillary network in a particular layer.

In the OCTA optic disc scan, vascular assessment in the superficial peripapillary retinal and choroidal layers helps diagnose glaucoma and detect disease progression (98). In glaucomatous eyes, the reduction of the vessel density in the superficial vascular plexus slab, currently the most widely used OCTA parameter, is more pronounced than in the deep retinal slabs. In contrast, the choriocapillaris localized in the parapapillary atrophy region, where the layers of the retina and RPE around the optic nerve become thinner, is used for the evaluation in the deep choroidal vessels (99). It avoids a limited resolution of the choroidal vessels due to the projection artifact that occurs when signals from the superficial retinal layers are projected onto the deeper layers. This well-known phenomenon results from the high variance related to strong blood flow of especially major vessels being cast onto the reflectance of more posterior structures, i.e., the “shadows” behind major vessels have variable intensity due to the high variability of their source.

Similarly, for the macular OCTA scans, the superficial retinal vessel density is regularly used to detect and monitor damage in glaucomatous eyes. Comparative studies have shown that a wider field of 6 x 6 mm scans is more sensitive in detecting glaucomatous changes than 3 x 3 mm scans (100). More recently, FAZ assessment has been used as an indicator of vascular reperfusion after surgery-induced IOP reduction in glaucomatous eyes (101). Thus, the RPC vessel density and the macular regions, the choroidal vasculature in the OCTA disc scan, and the FAZ evaluation in the macular scan may provide new insight into glaucoma management. OCTA is commonly used in the posterior pole but also to visualize iris neovascularization (102).

### 4.2 Decreased superficial vessel density helps detect glaucoma progression and may precede RNFL loss

The measurement of RPC density in ONH-centered scans and of the superficial vascular complex in macula scans both have excellent reproducibility and repeatability and display a similar primary open-angle glaucoma (POAG) diagnostic ability as other OCT parameters based on thickness values derived from reflectance differences (103). However, vessel density (ICC 0.823-0.871) values are more variable than cpRNFL (ICC 0.975) and GCC (ICC 0.995) thickness measured by OCT (104). This variability may reflect biological factors related to individual heterogeneity, including age, history of systemic vascular diseases, use of medications, and magnitude of glaucomatous vascular deficits, as well as characteristics susceptible to signal strength intensity reduction due to motion artifacts caused by relatively long imaging times and/or off-axis scan acquisition.

**Figure 3:**
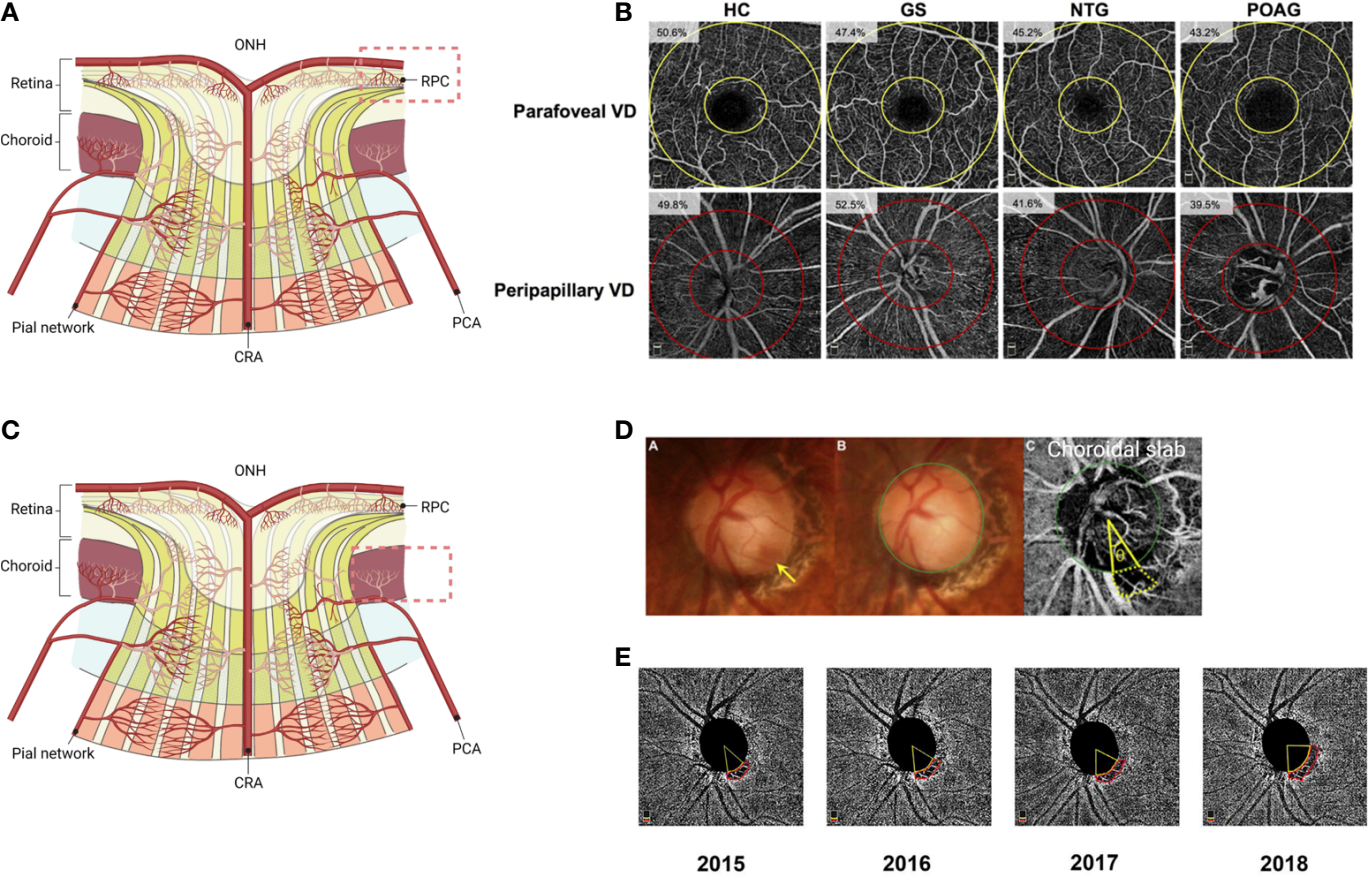
Utility of OCTA scans for glaucoma diagnosis and disease progression detection. **(A)** In glaucomatous eyes, peripapillary RPC vessel density and macular superficial vessel density decrease with increasing disease severity. **(B)** OCTA imaging of disease groups. Parafoveal and radial peripapillary capillary (RPC) vessel density measurements on en face OCTA images are shown. The parafovea is shown as the area between the two yellow circles on the angiograms centered on the fovea, while the RPC region is shown as the area between the two red circles on the angiograms centered on the optic disc. The measured vessel density is reported on each angiogram. HC, healthy controls; GS, glaucoma suspects; NTG, normal-tension glaucoma; POAG, primary open angle glaucoma; VD, vessel density. (Clinical Ophthalmology 2019:13 1935-1945. Originally published by and used with permission from Dove Medical Press Ltd.). **(C)** Microvascular dropout (MvD) in the deep choroidal layer derived from CA is a characteristic finding in glaucomatous eyes. **(D)** Determination of the circumferential extent and location of the parapapillary microvasculature dropout (MvD) topographically associated with disc hemorrhage (DH) locations (the yellow arrow indicates DH). Color photographs **(A, B)** and OCT angiography (OCT-A) deep-layer images **(C)** are shown. The green circle indicates the optic disc margin. The area demarcated by the yellow dotted lines is the MvD region. Lines are drawn from the disc center to where the MvD meets the optic disc margins; the angle (q) between these lines reflects the extent of MvD. (Ophthalmology 2018:125:1003-1013. Originally published by and used with permission from Elsevier Inc.). **(E)** En face choroidal vessel density map showing microvasculature dropout (MvD) area changes over 4-year follow-up in a primary open-angle glaucoma (POAG) eye. (American Journal of Ophthalmology 2022:241:130-138. Originally published by and used with permission from Elsevier Inc.) The figure was adapted with permission from Ref 105. **(A, C)** generated with BioRender (https://biorender.com/).

Prior studies have shown that the density of the RPCs around the ONH and of the superficial plexus in the macular region are significantly reduced in POAG eyes compared to control eyes as glaucoma severity increases (98) (**Figures 3A, B**). Notably, the vessel density measurements have lower floors compared to OCT thickness measurements, especially in the macular region (105), suggesting that the vessel density is a promising parameter for monitoring progression in advanced disease (particularly when VF mean deviation is worse than -14 dB).

Furthermore, in a longitudinal study assessing the risk of glaucoma progression, lower baseline macular and peripapillary vessel density was associated with a faster rate of cpRNFL loss (-0.11 μm/year per 1%; P<0.001, -0.66 μm/year; P=0.031, respectively) in mild to moderate glaucoma over an average follow-up of 27 months (106). Importantly, this association was independent of baseline cpRNFL thickness, suggesting that OCTA may provide additional information for risk assessment of glaucoma progression and prediction of the rate of disease worsening.

Uncovering the relationship between reduced vessel density in OCTA and corresponding RNFL loss may develop robust strategies for detecting pathological changes in the earliest stages of glaucoma. Interestingly, a cross-sectional study showed that the decreased vessel density was seen in the temporal sector corresponding to the papillomacular bundle in glaucomatous eyes with VF defects localized in one hemifield, despite intact RNFL thickness (107). This observation indicates that the superficial vessel density reduction may precede measurable cpRNFL changes. Thus, reduced superficial vessel density in glaucomatous eyes, which sometimes precedes RNFL thinning, helps clinicians assess disease progression from the earliest to advanced stages of glaucoma due to its low floor.

### 4.3 Choroidal deep-layer microvasculature dropout and FAZ

Deep-layer microvasculature dropout (MvD) is defined as the complete loss of choroidal vasculature derived from CAs in localized regions of the parapapillary atrophy (108) (**Figures 3C, D**). Recent studies have demonstrated that MvD in the choroidal slab is an actual perfusion defect confirmed by indocyanine green angiography and a signature feature in POAG eyes (99, 109, 110).

Recent studies have identified factors associated with the prevalence of MvD and associations between the MvD and structural and functional defects in POAG eyes. The majority of MvD worsened with increasing severity (99, 110–112). Interestingly, one study found that MvD was more frequent in POAG eyes with low pre-treatment baseline IOP (113). In keeping with this finding, a higher prevalence of MvD was shown in POAG eyes with disc hemorrhage (DH) and LC defects, which are found more frequently in normal-tension glaucoma (NTG), compared to those without MvD (99, 108, 114) (**Figure 3D**). Thus, MvD may be an observation that more reflects the pathogenesis of NTG. Furthermore, studies have shown that the presence of MvD is associated with a faster rate of cpRNFL thinning and VF progression (108, 115–117). In particular, a significant association between MvD and faster central VF progression has been reported (118). In addition, recent longitudinal studies have revealed that the area of MvD enlarged with disease progression and is associated with progressive cpRNFL thinning (117, 119) (**Figure 3E**).

Studies of primate fovea development suggest that foveal layer tissue is affected by FAZ size and IOP. As the FAZ takes shape during approximately 25 weeks of gestation, the fovea gradually deepens due in part to the influence of IOP. Subsequently, as anterior-posterior axial growth moves the inner retinal layer away from the fovea, cone photoreceptors migrate into the fovea and elongate. Importantly capillaries become attached to astrocytes and migrate outward from the fovea, resulting in a FAZ with a rounded configuration (120, 121). The macula is one of the most metabolically active human tissues and is specialized for central vision. The FAZ is sensitive to ischemic events and implicated in pathological processes. Previous studies have reported enlargement of the FAZ region in retinal ischemic diseases such as diabetic retinopathy (122, 123). An association between FAZ area size and structural/functional defects or IOP has also been reported in glaucoma (124).

A greater OCTA-measured FAZ area was associated with future cpRNFL and GCIPL thinning in POAG eyes over a mean longitudinal follow-up of 29 months (HR 1.73; P=0.036 and HR 2.6; P<0.001, per SD of FAZ area increase, respectively) (125). In addition, larger FAZ area was weakly associated with worse visual acuity (VA) in mild- to advanced stages of POAG eyes (R^2 =^ 0.11, P=0.035) (126). These findings indicate that FAZ enlargement is associated with a structural deficit in RGCs and affects central visual function as the disease progresses. Furthermore, FAZ size was reported to be associated with IOP. A prospective study evaluating FAZ size in glaucomatous eyes before and after surgery showed that reducing IOP decreases FAZ size (101). Although the large variability between individuals have been reported (127), FAZ size may be useful to capture intra-individual changes and a reversible decrease in FAZ size after IOP reduction may help assess vascular reperfusion after glaucoma surgery and may lead to recovery of macular RGC function.

## 5 Papillomacular bundle and QoL

### 5.1 Papillomacular bundle defect is associated with vision-related QoL in glaucoma

**Figure 4:**
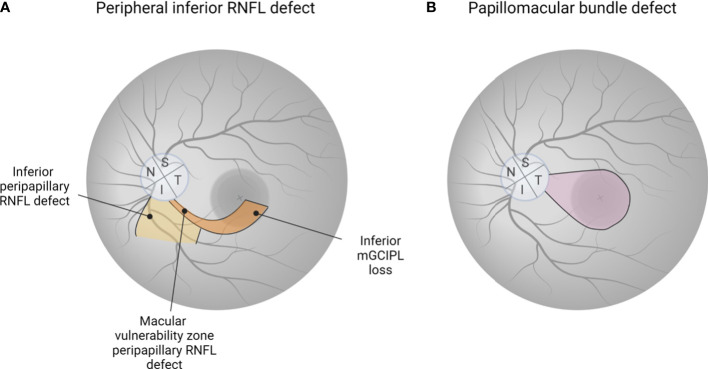
The importance of detecting glaucomatous eyes with preferential damage to papillomacular bundle. **(A)** Peripheral inferior RNFL defect is commonly observed as an early sign of glaucoma. Macular damage in glaucoma is more common than previously thought, with the “macular vulnerability zone” in the inferotemporal macular region, where nerves are densely located, being the most vulnerable. **(B)** There is growing recognition that papillomacular bundle defects, even in the early stages of glaucoma, can occur. Since loss of papillomacular bundle is directly related to impairment of central vision, leading to a reduced quality of life, new tools such as RNFL optical texture analysis are needed to detect the region-specific defect at an early stage. **(A, B)** generated with BioRender (https://biorender.com/).

Papillomacular bundle refers to the axons arising from the RGCs surrounding the fovea. While peripheral VF defects are commonly observed as an early sign in glaucoma, it is increasingly recognized that central VF defects and corresponding macular RNFL defects may also occur in the early stages of the disease (128, 129) (**Figures 4A, B**). The mechanisms underlying the differential susceptibility of the papillomacular bundle and the peripheral areas in glaucomatous eyes remain unclear. However, it is known that the RGC axons of the papillomacular bundle have a smaller caliber and require a relatively more significant amount of ATP to maintain axonal transport than larger fibers (130). Given the energy decline and axonal transport deficit of RGC axons in the early stages of glaucoma (131–134), this anatomic vulnerability may limit mitochondrial bioenergetic reserve capacity and proportionately increase production of reactive oxygen species, similar to mitochondrial optic neuropathies such as Leber’s hereditary optic neuropathy and autosomal dominant hereditary optic neuropathy, which are characterized by preferential loss of RGCs in the papillomacular bundle (135). Prior studies have shown that macular damage can lead to severe decline in the vision-related QoL (136, 137). However, it is also possible that the papillomacular bundle of axons and the foveal RGCs are ultimately injured by the same processes, perhaps within the LC, as other, more peripheral RGCs and their axons.

### 5.2 Imaging evaluation of the macula and visual acuity

Patients with glaucoma experience impairments in daily activities critically important to independent living such as driving and reading (138). Objective imaging evaluation of the macula is essential due to its high concentration of RGCs, which are required for normal central vision. Hood et al. reported that macular damage in glaucoma may be more prevalent than previously thought and that the inferior macula appears to be more susceptible to damage than the superior macula. Their results support the “crowding hypothesis,” which posits that the inferior fibers are more susceptible to damage as they enter the more densely populated inferotemporal area of the nerve, referred to as the “macular vulnerability zone” (139) (**Figure 4A**). This likely also reflects that in the vast majority of eyes, the fovea is offset below the horizontal midline through the ONH.

While VF loss is a distinctive characteristic of glaucoma, VA is also an important functional parameter that affects the vision-related QOL (140). Kim et al. investigated the correlation between retinal thicknesses and VA, and found a stronger association in eyes with advanced glaucoma (141). Another study examined the relationship between VA and OCTA parameters, and found the higher area under the curve (AUC) values on deep macular vessel density (AUC=0.740) and deep nasal grid vessel density (AUC=0.748) with the cut-off values of VA<20/25 in advanced glaucoma eyes (142). Moreover, Wu et al. reported that some macular OCT and OCTA parameters were associated with VA in moderate to advanced glaucoma, with the superior hemifield performing better than the inferior hemifield, but not in early glaucoma (126). Most OCT and OCTA parameters showed modest discriminatory power for glaucoma eyes with decreased VA, with parafoveal GCC showing the best overall discrimination (126, 143).

### 5.3 New technology to visualize the papillomacular and papillofoveal bundles

Contrary to the widely held belief that the fovea and macula are preserved until the late stages of glaucoma, a recent study showed that papillofoveal and papillomacular bundle defects may be affected in early stages of glaucoma (129) (**Figure 4B**). RNFL optical texture analysis is a technique recently introduced by Leung et al. and reported to be useful in the detection of papillomacular and papillofoveal defects with excellent accuracy, reproducibility, and sensitivity (144). In their study including 204 early glaucomatous eyes of 171 consecutive enrolled patients, 72% and 17% showed RNFL defects involving the papillomacular and papillofoveal bundles, respectively (129). Evolving new technologies could alter the paradigm of the evaluation and management of glaucoma.

## 6 Imaging within the ONH

### 6.1 Glaucomatous optic disc cupping: alterations in the laminar and prelaminar regions

**Figure 5:**
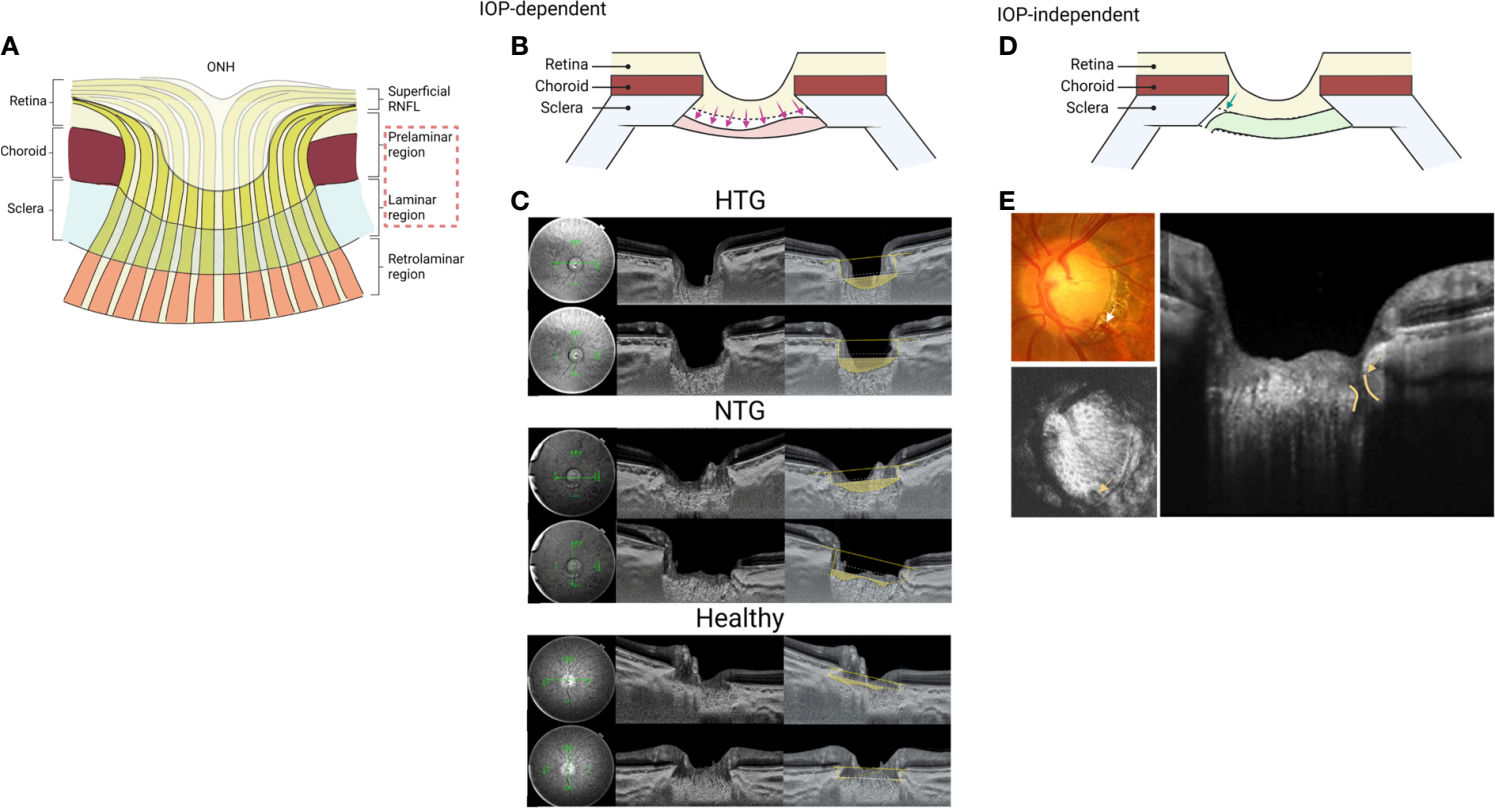
Findings of lamina cribrosa imaging in glaucoma. **(A)** Glaucomatous optic disc cupping consists of two components: changes in the laminar and prelaminar regions. Glaucomatous cupping reflects remodeling and damage of the laminar connective tissues at the level of laminar region and progressive loss of RGC axons in the prelaminar region. **(B)** Lamina cribrosa operates biomechanically in an IOP-dependent manner. Three OCT-derived lamina cribrosa parameters: lamina cribrosa depth, anterior laminar cribrosa insertions, and lamina cribrosa curvature are widely used to mirror IOP-related glaucomatous changes. **(C)** Representative swept-source optical coherence tomography (SS-OCT) B-scans of optic discs in high-tension glaucoma (HTG), normal-tension glaucoma (NTG) and healthy eyes. Horizontal and vertical optic disc scans of HTG, NTG and healthy eyes. The area shaded with yellow depicts the degree of posterior bowing of the lamina cribrosa according to the level of anterior laminar insertion depth (white solid line). The figure was adapted with permission from PLoS ONE 2016: 11: e0162182. **(D)** In contrast, focal lamina cribrosa defects are characteristic of glaucomatous eyes with high myopia, loss of choriocapillaris, and DH. **(E)** Lamina cribrosa SS-OCT images in a glaucoma patient with optic disc hemorrhage. Optic disc hemorrhage was detected in the inferotemporal area of the optic disc by color optic disc photography. En-face image of the optic disc at the level of lamina cribrosa. The radial scan image from the SS-OCT showed the focal lamina cribrosa defect corresponding with optic disc hemorrhage. The figure was adapted with permission from Investigative Ophthalmology & Visual Science 2016: 57:899-907. **(A, B, D)** generated with BioRender (https://biorender.com/).

Glaucomatous optic disc cupping consists of two components: changes in the laminar and prelaminar regions (145) (**Figure 5A**). Laminar cupping results from connective tissue failure and remodeling; chronic exposure to IOP, a critical biomechanical load even if not statistically elevated, or low intracranial pressure that increases sensitivity to IOP causes the LC of susceptible eyes to become displaced and bowed posteriorly. This can reflect compression and active remodeling of the LC microarchitecture, and manifest clinically in part as enlargement and elongation of the pores through which axon bundles pass, and ultimately as the well known “excavation” of advanced glaucomatous cupping. The lateral insertions of the LC can also undergo remodeling such that there is increasing likelihood of its collagen fibrils being inserted into the pia mater rather than peripapillary sclera. The prelaminar aspects of glaucomatous ONH cupping represent partly compression driven by LC changes, but also by progressive loss of axons from the prelaminar neural rim tissues, the latter of which occurs in later stages of all optic neuropathies. Thus, glaucomatous cupping reflects remodeling and damage of the laminar connective tissues and progressive loss of RGC axons (146). Given the central pathology of the LC in glaucoma, assessing glaucomatous changes in the LC *in vivo* using OCT could provide crucial insights into the progression and detection of glaucoma risk factors. Indeed, new technologies and techniques such as SS-OCT and enhanced depth imaging improved *in vivo* imaging of deeper ocular structures, thus allowing research on the role of the LC in glaucoma.

### 6.2 LC operates biomechanically in an IOP-dependent manner

The LC plays a prominent role in the pathogenesis of glaucoma. With the advent of the enhanced depth imaging SD-OCT and SS-OCT, *in vivo* evaluation of the LC deformation in glaucoma eyes has generated considerable interest. Three OCT-derived LC parameters: LC depth, anterior LC insertions, and LC curvature mirror IOP-related glaucomatous changes (**Figures 5B, C**). LC depth is commonly defined as the distance from the line connecting both ends of BMO to the anterior surface of the LC (147–151). Previous studies showed that the LC depth measurement was significantly increased in eyes with glaucoma. In addition, increased LC depth was associated with younger age, higher untreated IOP, a thinner cpRNFL, peripherally located central retinal vein trunk, and disease severity as determined from VF damage (151). Moreover, a longitudinal study demonstrated that LC depth in glaucomatous eyes decreased significantly three months after IOP-lowering surgery, and the rate of change correlated with the percent change in IOP and worse VF mean deviation, highlighting the dynamic nature of the structure and mechanical environment of the LC and surrounding tissues remodeling (147).

The LC insertions are commonly determined by the vertical distance between the anterior lamina insertion to the scleral wall and the reference plane connecting the BMO (152, 153). Previous studies showed that the anterior LC insertions were located more posteriorly in eyes with glaucoma than in eyes of healthy controls.

Previous studies reported that the LC curvature index, defined as the difference between the mean LC depth and the anterior LC insertion depth, was significantly higher in patients with glaucoma than in healthy controls (153–155). Higher curvature was associated with male gender and higher IOP, while the glaucoma severity had no association (153). Interestingly, a prospective study found that a higher baseline in the laminar curvature index was strongly associated with future glaucomatous VF defect progression (154).

In keeping with these findings, a cross-sectional study revealed that LC depth and the LC insertions were located more posteriorly, and the curvature of the lamina was higher in eyes with high-tension glaucoma (HTG) than in NTG (149), further supporting the idea that the LC deformation is primarily caused by mechanical stress in IOP-dependent manner (**Figure 5C**). These findings collectively support the histologic changes in glaucomatous eyes where chronic elevated IOP causes progressive LC deformation and posterior migration with loss of RGC axons.

### 6.3 Anterior focal LC defects are particularly seen in glaucomatous eyes with high myopia, choroidal MvD, and DH

Unlike LC depth, insertions, and curvature, focal LC defects are a particular characteristic of glaucomatous eyes with high myopia, loss of choriocapillaris, and DH (**Figures 5D, E**). A focal LC defect is defined mainly as an irregularity on the anterior surface of the lamina that invades the normal smooth curved contour of the U- or W-shaped lamina with at least a diameter of 100 µm and a depth of 30 µm (156–159). The frequency of the focal LC defects was reported to be significantly higher in glaucomatous eyes than in normal eyes, with 50% of glaucomatous eyes having focal LC defects, compared to 0.03% of control eyes (159). Furthermore, two previous studies examining the presence of focal LC defects in patients with high myopia with or without glaucoma found that focal LC defects were significantly more frequent (42-54%) in myopic eyes with glaucoma compared to 2-23% of myopic eyes without glaucoma (159, 160). Thus, focal LC defects may provide helpful information in differentiating glaucoma in highly myopic eyes, where glaucoma can be otherwise difficult to detect.

One cross-sectional study found that 80% of glaucomatous eyes with focal LC defects had VF defects in the hemisphere corresponding to the most extensive focal LC defect. In contrast, the remaining 20% of the glaucomatous eyes had no VF defect (159), suggesting that damage to the LC precedes the significant VF defect. Further longitudinal studies are needed to better understand the spatial and temporal correlation between the presence of focal LC defects and VF defects.

In addition, recent studies using OCTA demonstrated that glaucomatous eyes with focal LC defects have more choroidal MvD (99, 161), indicating that these two glaucomatous components may be spatially and temporally correlated. Furthermore, focal LC defects appeared more frequently in glaucomatous eyes with DH than in glaucomatous eyes without DH and were located more proximally to the DH. In studies by Kim and Park, focal LC defects were found in 81% of eyes with DH compared to 40% of eyes without DH, and the size of focal LC defects-associated DH was greater and more proximally located (157, 158) (**Figure 5E**). MvD and DH are risk factors for glaucoma progression, notably in NTG. Given that histopathologic findings of focal nerve fiber disruption are more prominent within the optic nerve in NTG eyes than in POAG eyes, the region of focal LC defects may be an active site where axonal damage of RGCs and breakdown of the prelaminar capillaries and capillary-containing laminar beams.

### 6.4 LC thickness and LC architecture changes in glaucoma

The LC thickness is defined as the distance between the anterior border, where the laminar pores become visible, and the posterior margin, where the laminar pores are invisible on en face images, or the distance between the anterior and posterior margin of the highly reflective area visible within the ONH on cross-sectional B-scan SS-OCT images (162–164). Numerous studies have reported that glaucomatous eyes have significantly thinner LC compared to healthy eyes, especially in advanced disease. It should be noted, however, that due to its variability, visualization of the posterior border of LC using SS-OCT is still challenging and further technological advances are needed for consistent whole LC imaging with higher resolution. In addition, LC architecture changes in glaucoma, such as lamina pore size and shape changes have been observed using SS-OCT (165, 166). Although the lamina pores are challenging to visualize, previous studies found that the lamina pores are generally elongated, have greater size variability, and increased tortuosity in glaucoma. The laminar beams and pore size also decreased with increasing distance from the central retinal vein trunk (167). This decrease was more pronounced in eyes with glaucoma, suggesting remodeling of the laminar beams in glaucoma (45).

### 6.5 Vascular changes within the ONH in glaucoma

Most of the blood supply to RGC axons within the LC is not directly proximal, rather, capillaries are located within the connective tissue beams, thus, separated from the axon bundles by collagenous tissue. Therefore, many of the axons within the LC, require diffusion of oxygen and other nutrients from the laminar capillaries through the surrounding beam structure, unlike other regions of the ONH or orbital optic nerve. The IOP-induced stress and strain on the peripapillary sclera may compress the penetrating branches of the CAs and perhaps also the laminar capillaries resulting in perfusion abnormalities, notwithstanding that the ONH blood flow is controlled by autoregulation. Indeed, it has been shown that IOP-related stress can cause compression of capillaries within the peripapillary choroid, the prelaminar ONH tissue, and within the LC in the monkey experimental glaucoma model (47). In addition, a longitudinal study demonstrated that compromised blood flow during ocular hypertensive damage, which hinders oxygen and nutrient supply to energetically demanding RGCs, triggers neuronal dysfunction in a preclinical mouse glaucoma model (168). Furthermore, the geometry and stiffness of the connective tissue within the LC and the cellular reactivity of astrocytes, microglia and other immune cells, may also enhance vascular and neuronal vulnerability to IOP, even at a normal IOP range (145). Lastly, IOP-independent factors, such as fluctuations in systemic blood pressure and vasospasm in the retrobulbar PCA region, may also affect ONH blood flow (169–171)

As mentioned above, the direct shadowing effect and the flow projection artifact make it difficult to detect deep-layer blood flow perfusing the LC using OCTA. Despite such limitations in the visibility of deep vasculatures, one initial study using OCTA reported that POAG eyes had decreased vessel density and flow index, defined as the average decorrelation values within ONH, compared to control eyes (172). Similarly, in a study using laser speckle flowgraphy, which has been shown to reflect critical CAs-derived ONH blood flow nourishing the LC (173), the ONH blood flow was already reduced in eyes with preperimetric glaucoma, the earliest stages of the disease (174). Furthermore, a follow-up longitudinal study demonstrated that the reduced baseline ONH blood flow was associated with future VF progression in preperimetric glaucoma eyes, suggesting that laminar capillary flow is impaired from the earliest stages of glaucoma and precedes functional VF deficits (175). However, further improvement in imaging techniques is needed to resolve these issues.

## 7 Future perspectives of the OCT/OCTA in glaucoma

OCT and OCTA are essential tools for the diagnosis and monitoring of disease progression of glaucoma (**Table 1**); however, many significant challenges remain including prediction of disease progression or risk, segmentation errors (more frequent and significant in myopia and/or other concurrent morbidities), variations in image quality due to technical limitations, and artifacts due to relatively long duration of some scan types/imaging protocols.

**Table 1:** Summary of OCT/OCTA parameter characteristics associated with glaucoma.

OCT parameter	Characteristics
cpRNFL	• The most widely studied parameter that can be assessed with OCT
	• Average and inferior-sector thickness are the most sensitive parameters in both the diagnosis and progression detection of glaucoma
Macula (GCIPL, GCC)	• May be more senstive in detecting progression in advanced glaucoma
	• Papillomacular bundle defect is associated with vision-related QoL in glaucoma • Potential usefulness in reducing the impact of segmentation errors in myopic eyes
ONH (BMO-MRW, Beta-zone PPA, LC-related parameters)	• BMO-MRW has been proposed as a sensitive, reproducible measurement of the ONH for early glaucoma detection.
	• Beta-zone PPA has been proposed for improved progression detection
	• LC depth, anterior LC insertions, and LC curvature mirror IOP-related glaucomatous changes
	• Anterior focal LC defects are particularly observed in glaucomatous eyes with high myopia, MvD, and DH
OCTA parameter	
Superficial vessel density	• Superficial vessel density, especially in the macular region, may be useful for monitoring progression in advanced disease due to its low floor
Choroidal MvD	• The presence and enlargement of MvD are associated with DH and LC defect and particularly useful in assesing progression in glaucomatous eyes with central VF defects
FAZ size	• Associated with a future structual cpRNFL and GCIPL thinning and IOP

The ideal way to detect glaucoma progression is to be able to determine not only whether the disease is progressing, but also predict the worsening of the disease. The rate of glaucoma deterioration can vary widely from patient to patient. That is, most patients progress relatively slowly, while others have progressive disease that can worsen rapidly and ultimately result in blindness or significant QOL impairment without appropriate timely therapeutic intervention. Recent studies demonstrate a large myriad of potential applications of artificial intelligence (AI) in glaucoma imaging toward establishing precision medicine, from prediction of progressing eyes to proper segmentation detection (the details are well summarized in the review by Ma et al. (176)). For example, machine learning and deep learning models, such as gated transformer networks, and mathematical models including elastic net logistic regression, can predict VF progression from longitudinal OCT measurements in glaucomatous eyes with reasonable accuracy (177). Further refinement of the proposed model could help predict functional disease deterioration promptly with less burdensome structural testing, which would be helpful for clinical decision-making. Moreover, using an AI approach based on ONH morphological changes and VF impairment patterns characteristic of glaucoma allows the identification of POAG phenotypes (178). Such AI-based objective clustering will provide new insights into improving structure-function relationships and subsequent VF worsening prediction in POAG phenotypes, ultimately leading to precision medicine.

The presence of myopia and retinal pathology could lead to inaccurate segmentation and result in higher variability (179). Utilizing updated, validated software may confer an advantage to reduce segmentation errors. Furthermore, recent studies have demonstrated that unsegmented OCT as an input for deep learning models, an AI-assisted algorithm, has merits in detecting glaucoma and predicting VF damage (180, 181).

The utilization of *in vivo* imaging techniques with sufficient resolution to visualize cellular structures of the human retina may prove advantageous in the early detection and progression monitoring of disease. The pathogenic alterations originate from functional and structural changes at the cellular level, well before any decline in vision can be identified. Adaptive optics (AO), a technology initially developed in astronomy, has been employed to achieve microscopic lateral and axial resolution by combining it with OCT into AO-OCT (182), with applications to study glaucoma at cellular resolution scale in the living eye (183). Although the field of view is currently limited to the isoplanatic area, if stability and a broader range of imaging can be attained in the future, it could be useful in clinical practice, such as clear visualization of the microstructure in the LC and of the outermost off-sublamina layer in the retinal IPL, where histological changes first occur in experimental glaucoma models (184, 185). In conjunction with current OCT/OCTA techniques, the visible-light OCT, using shorter (visible) wavelengths, is also expected to furnish additional information, such as stratification analysis of the IPL and oximetry of retinal vessels (186, 187). The visible-light OCT has some limitations, including subjects’ fixation error and attenuation of imaging sensitivity due to visible light. Nevertheless, early disease identification through such advanced imaging methods may facilitate personalized care with improved sensitivity compared to conventional diagnostic techniques.

Despite the release of updated software with enhanced image processing by various companies, longitudinal evaluation for OCTA remains problematic owing to low reproducibility and OCTA-specific artifacts. One study showed that more than 30% of the OCTA images had poor quality, despite a smaller proportion having severe glaucoma, which is typically associated with low image quality (188). The implementation of new machines capable of capturing images in a shorter time frame, or hardware that can perform rescanning of areas with motion artifacts, may permit more reproducible measurements.

Thus, advances in hardware and software sophistication are eagerly anticipated in the near future to benefit the management of glaucoma patients by improving segmentation errors, artifacts, image quality, and by enabling quantitative assessment of novel imaging targets.

## 8 Conclusion

Recent advances in imaging instruments have allowed for a more comprehensive and objective understanding of glaucoma. This is of particular importance, as glaucoma is a multifactorial chronic eye disease that is characterized by progressive damage to the optic nerve, leading to vision loss. The use of advanced imaging modalities such as OCT and OCTA, is essential in order to accurately diagnose glaucoma in its early stages and to monitor the progression of the disease.

Diagnostic imaging equipment will take on an even greater role for the management of glaucoma in the clinical setting. In the future, it is also desirable to improve methods to make imaging more convenient and expeditious for clinicians to use, in light of the increase in volume resulting from an aging population and the advancement of telemedicine. In conclusion, recent imaging modalities are promising tools for glaucoma, leading to clinical and economic benefits.

## Author contributions

YS, TN, JJ, AP, and BF wrote the manuscript. All authors contributed to the article and approved the submitted version.
